# Arteriovenous Malformation of the Uterus in a 41-Year-Old Woman Presenting as Uterine Bleeding

**DOI:** 10.7759/cureus.23646

**Published:** 2022-03-30

**Authors:** Thomas Cotter, Ahmed Arfa, Pramila Moideen, Asad Ullah, Intisar Ghleilib

**Affiliations:** 1 Pathology, Augusta University Medical College of Georgia, Augusta, USA; 2 Pathology and Laboratory Medicine, Augusta University Medical College of Georgia, Augusta, USA

**Keywords:** cesarean section (cs), myometrium, uterine bleeding, vascular lesion, arteriovenous malformations

## Abstract

An arteriovenous malformation (AVM) is a vascular lesion most frequently encountered in the brain, lungs, colon, and soft tissues of the extremities. However, rarely, an AVM may develop in the uterus, where it can cause abnormal and even life-threatening uterine bleeding. Here, we present the case of a 41-year-old G6P6 woman with abnormal uterine bleeding which resulted in a hemoglobin level of 10.2 g/dL. On gross examination, the uterus was enlarged measuring 17.5 cm x 12.0 cm x 10.0 cm, with a pronounced globoid appearance and bogginess on palpation. The cut surface was hemorrhagic and notable for numerous tortuous dilated spaces of variable sizes. These hemorrhagic, cavernous spaces were grossly apparent throughout the entire myometrium, but were found to be most prominent in the lower uterine segment of the anterior wall. Microscopic examination revealed an admixture of malformed vasculature comprising arteries, venules, and capillaries. The vessels showed prominent dilation and tortuosity with abrupt variation in the thickness of the media and elastic lamina, as highlighted by Von Gieson stain. Unlike in many other organ systems where AVMs are often considered congenital lesions, uterine AVMs are more often acquired lesions that develop following iatrogenic uterine trauma, namely cesarean section or curettage. Upon review of our patient’s history, her final delivery was via cesarean section, after which she developed abnormal uterine bleeding. We present this case as a reminder to consider uterine AVM in cases of abnormal uterine bleeding, as it may be easily overlooked by even the most experienced pathologist.

## Introduction

The abstract of this article was previously presented at the College of American Pathologists (CAP) meeting, Chicago, September, 25-28, 2021.

An arteriovenous malformation (AVM) is defined as an abnormal connection between an artery and a vein, which bypasses the usual intervening capillary network [1]. This vascular lesion is most frequently seen in the brain, lungs, colon, and soft tissues of the extremities, but is rarely observed in the uterus [2]. Owing to sparse case reports, the exact prevalence of uterine AVMs is unknown [3]. They often develop and are present in women during their reproductive years, although a congenital variant has also been identified. The correct identification and diagnosis of uterine AVMs are essential, as missed diagnosis or improper management can cause life-threatening bleeding [1]. Herein, we present the case of a 41-year-old woman with vaginal bleeding of unknown etiology.

## Case presentation

**Figure 1:**
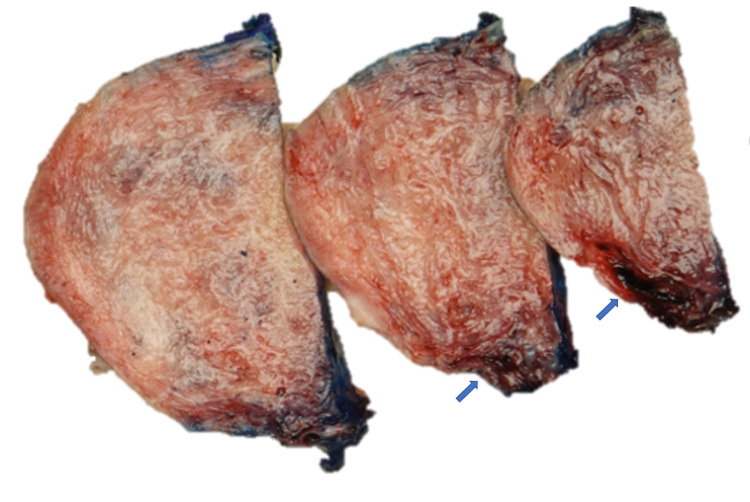
Gross photographs of arteriovenous malformation of the uterus. A grossly dilated, tortuous, hemorrhagic vascular lumina penetrating the myometrium (arrow).

Our patient was a 41-year-old, G6P6006, with a history of abnormal uterine bleeding, uterine fibroids, cyclical pelvic pain, and morbid obesity (body mass index, BMI: 42.1). All her pregnancies resulted in live births; the first five were delivered vaginally, with the sixth delivered via cesarean section followed by tubal ligation. She denied any history of curettage, spontaneous abortion, or molar pregnancies. She presented to us with the chief complaint of menorrhagia lasting five to seven days, concurrent with the passage of large clots and severe cramping. Her physical examination showed a 10-week sized anteverted uterus with frank blood at the external cervical os. She had previously been diagnosed with large uterine leiomyomata and had undergone a prior endometrial biopsy. Endometrial biopsy revealed a weakly proliferative endometrium, which was negative for malignancy or hyperplasia. Imaging revealed a large uterine mass that was perforated and perfused by feeder’s vessels along with an irregular cervical mass heavily interlaced with uterine vessels. Further imaging revealed no evidence of metastatic disease deposits in the abdomen or the chest. Initially, endometrial ablation was considered but decided against because of the size and extent of the fibroids. Thus, the patient ultimately underwent an elective total hysterectomy. An intraoperative frozen section was performed, which revealed a spindle cell lesion with no overt malignancy. The uterus was subsequently subjected to a pathological evaluation. Gross findings revealed a 17.5 cm × 12.0 cm × 10.0 cm, 685 g uterus. The cut surface revealed prominent, tortuous, dilated spaces that were grossly hemorrhagic (Figure 1).

**Figure 2:**
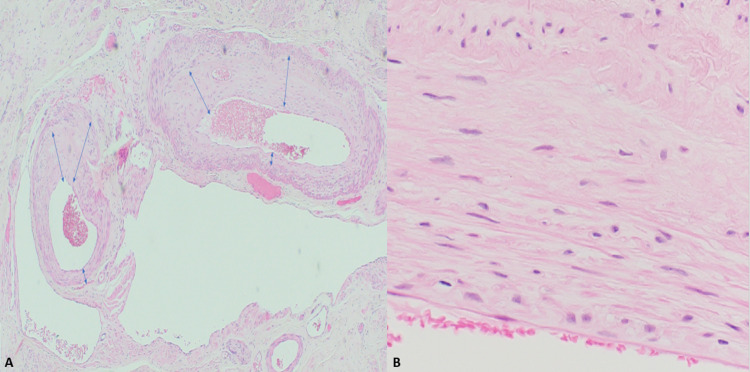
Hematoxylin and eosin: (A, 20x) Multifocal aggregates of large caliber, irregular vasculature seen in the myometrium. (B, 400x) The cells in the vessels wall has bland cytology with no atypia.

**Figure 3:**
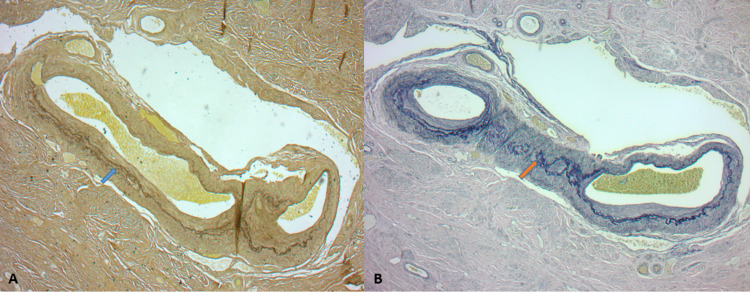
(A & B) Verhoeff-van Gieson elastin stain: highlighting the disrupted, unevenly reduplicated internal elastic lamina traversing the variably sized, or even absent tunica media (arrow). This juxtaposition illuminates the hybrid artery and venous nature of the aberrant vessels.

Microscopically, the lesion revealed multiple, dilated irregular vessels penetrating the myometrium. The cells comprising these aberrant vessels were bland and largely monomorphic, lacking cytological atypia, mitoses, or necrosis. These unremarkable nuclear findings helped eliminate leiomyosarcoma and several vascular neoplasms from the differential diagnoses. An additional key finding was the discohesive elastic layers which were illuminated with Von Gieson elastin stain. This stain highlighted abrupt variations in the thickness of the tunica media and elastic lamina within the same cross-section of individual vessels, unmasking their hybrid nature (Figures 2-3).

The structural abnormalities, concurrent with the regular cytology of these vessels, led to a diagnosis of AVM of the uterus.

## Discussion

Uterine AVMs were first reported in 1926 [1, 4]. These are rare entities with an unknown prevalence, with less than 100 cases documented in the literature [4-5]. An AVM of the uterus is most commonly a fistulous connection between the branches of the uterine artery and the myometrial venous plexus, which bypasses the normal intervening capillary network [1]. Consequently, highly pressurized blood flows from the artery into the vein without dissipation through the capillary plexus, often resulting in profuse bleeding. One study showed that up to 50% of patients with acquired uterine AVM required blood transfusion secondary to anemia [6].

Acquired uterine AVMs most commonly develop as a result of an iatrogenic insult to the uterus, followed by an aberrant attempt at physiologic repair. One review of 54 patients with acquired uterine AVM showed that 96% had a history of cesarean section, dilatation, curettage, myomectomy, or placement of an intrauterine device [3]. In terms of pathogenesis, if the uterine vascular plexus is transected during an invasive procedure, surgical packing to achieve hemostasis may artificially shunt blood while concurrently approximating the newly created artery and venous margins. If the healing process begins when these larger vessels are juxtaposed with no intervening capillary plexus, a fistulous connection may be established upon resumption of physiological blood flow [3-4]. Congenital AVMs are believed to result from defective differentiation in the primitive fetal capillary plexus, often presenting in early menarche, as hormonal changes induce proliferation and increase blood flow [3].

The consequences of these insidious lesions can be profound. Clinically, AVMs often present as abnormal to heavy vaginal bleeding, accounting for up to 4.5% of all genital and intraperitoneal hemorrhage, but less commonly present with polyuria, dyspareunia, pelvic pain, or congestive heart failure [5, 7-8]. Historically, the diagnosis was made by a pathologist after a hysterectomy [9]. Today, diagnosis hinges on careful review and scrutiny of imaging studies, with digital subtraction angiography being the gold standard [10]. Importantly, if the diagnosis is attempted via hysteroscopy or dilatation and curettage, mechanical prodding can cause a fragile AVM to rupture, resulting in life-threatening hemorrhage [3]. Akin to its former diagnostic role, hysterectomy is the gold standard of treatment for suspected uterine AVM as well [4]. Today, while this remains the most efficacious therapy, in women looking to preserve fertility, uterine transarterial embolization is the most widely utilized approach, with a success rate of 91% after the second embolization treatment [3]. Various medications, including oral norethisterone, methotrexate, misoprostol, and methylergometrine, can also be used in less unstable and clinically deleterious lesions. Every non-excisional approach aims to curtail blood flow at the focus of the fistula or nidus, thus abating hemorrhage [1].

Grossly, uterine AVMs may appear as floridly hemorrhagic, dilated, large-caliber vessels, which are much more conspicuous than normal blood-laden vessels. They can display grossly tortuous luminal trajectories with varying vessel wall thicknesses, mimicking hemorrhagic cystic cavitation [5, 9].

Microscopically, uterine AVMs show proliferation of bland, spindled endothelial cells that lack cytologic atypia [9, 11]. These morphologically unremarkable cells comprise structurally remarkable vessels, which can be further identified as hybrids. These hybrids may contain an amalgam of arterial and venous components in the same cross-section, such as an internal elastic lamina, but no prominent tunica media. Verhoeff-van Gieson stain illuminates key diagnostic patterns, such as irregular, discohesive, reduplicating elastin fibers traversing variably sized vessel walls [9, 12].

## Conclusions

Uterine AVMs can be present at birth or are more likely to develop after rote, ostensibly innocuous gynecological procedures. Given their propensity to bleed, a non-invasive diagnosis by the clinical team is ideal. However, hysterectomy remains the definitive treatment for idiopathic abnormal uterine bleeding. The clinical-pathological diagnosis is important to rule out aggressive uterine malignancies, e.g. leiomyosarcoma, and rarely angiosarcoma.

## References

[1] Giurazza F, Corvino F, Silvestre M (2021). Uterine arteriovenous malformations. Semin Ultrasound CT MR.

[2] Laakso A, Dashti R, Juvela S, Niemelä M, Hernesniemi J (2010). Natural history of arteriovenous malformations: presentation, risk of hemorrhage and mortality. Acta Neurochir Suppl.

[3] Yoon DJ, Jones M, Taani JA, Buhimschi C, Dowell JD (2016). A systematic review of acquired uterine arteriovenous malformations: pathophysiology, diagnosis, and transcatheter treatment. AJP Rep.

[4] Hashim H, Nawawi O (2013). Uterine arteriovenous malformation. Malays J Med Sci.

[5] Hammad R, Nausheen S, Malik M (2020). A case series on uterine arteriovenous malformations: a life-threatening emergency in young women. Cureus.

[6] Peitsidis P, Manolakos E, Tsekoura V, Kreienberg R, Schwentner L (2011). Uterine arteriovenous malformations induced after diagnostic curettage: a systematic review. Arch Gynecol Obstet.

[7] Koyalakonda SP, Pyatt J (2011). High output heart failure caused by a large pelvic arteriovenous malformation. JRSM Short Rep.

[8] O'Brien P, Neyastani A, Buckley AR, Chang SD, Legiehn GM (2006). Uterine arteriovenous malformations: from diagnosis to treatment. J Ultrasound Med.

[9] Lollie TK, Raman SS, Qorbani A, Farzaneh T, Moatamed NA (2020). Rare occurrence of uterine arteriovenous malformation clinically mimicking a malignant growth: a critical reminder for pathologists. Autops Case Rep.

[10] Khan S, Saud S, Khan I, Achakzai B (2019). Acquired uterine arteriovenous malformation following dilatation and curettage treated with bilateral uterine artery embolization: a case report. Cureus.

[11] Capmas P, Levaillant JM, Teig B, Fernandez H (2013). Uterine arteriovenous malformation involving the whole myometrium. Ultrasound Obstet Gynecol.

[12] Piccinin MA, Schwartz J (2021). Histology, Verhoeff Stain. https://www.ncbi.nlm.nih.gov/books/NBK519050/.

